# ALNCR.TNM as a Novel Prognostic Tool for Cancer Survival

**DOI:** 10.1002/jcsm.70339

**Published:** 2026-08-18

**Authors:** Heyang Zhang, Guotian Ruan, Zelin Chai, Chong Li, Jinyu Shi, Chenan Liu, Shutian Zhang, Peng Li, Shengtao Zhu, Hanping Shi

**Affiliations:** ^1^ Department of Gastroenterology, Beijing Friendship Hospital, National Clinical Research Center for Digestive Diseases, Beijing Digestive Disease Center, Beijing Key Laboratory for Precancerous Lesion of Digestive Diseases Capital Medical University Beijing China; ^2^ Department of Gastrointestinal Surgery/Department of Clinical Nutrition, Beijing Shijitan Hospital Capital Medical University Beijing China; ^3^ Key Laboratory of Cancer FSMP for State Market Regulation Beijing China; ^4^ Department of General Surgery, Beijing Friendship Hospital, State Key Lab of Digestive Health, National Clinical Research Center for Digestive Diseases Capital Medical University Beijing China; ^5^ State Key Laboratory of Virology, College of Life Sciences Wuhan University Wuhan China; ^6^ Department of Oncology Affiliated Dazu's Hospital of Chongqing Medical University Chongqing China

**Keywords:** ALNCR.TNM, prognosis, systemic inflammation

## Abstract

**Background:**

Systemic inflammation is associated with cancer prognosis, but commonly used inflammatory indices show limited discrimination across heterogeneous malignancies. We developed and validated a novel inflammatory burden index, ALNCR, and tested whether integrating ALNCR with TNM stage improves overall survival (OS) prediction.

**Methods:**

We analysed 6374 adults with cancer from the multicentre INSCOC cohort (2013–2021), randomly split into a training cohort (*n* = 4464) and internal validation cohort (*n* = 1910), and an external validation cohort from Fujian Cancer Hospital (*n* = 759). Female proportions were 39.0%, 41.2% and 32.0%, and median ages were 60, 60 and 58 years, respectively; Stage IV disease accounted for 45.4%, 45.8% and 64.8%. ALNCR was defined as (albumin × lymphocyte) / (neutrophil × C‐reactive protein). Discrimination was assessed using C‐index and time‐dependent AUC, with comparisons to NLR, PLR, CAR, SII and PNI. A combined model (ALNCR.TNM) used ALNCR (cutoff 3.21) and TNM stage (I/II vs. III/IV); incremental value was quantified by net reclassification improvement (NRI) and integrated discrimination improvement (IDI).

**Results:**

ALNCR showed the highest discrimination for OS among evaluated indices (C‐index 0.645 [95% CI 0.631–0.658] in training; 0.666 [0.645–0.686] in internal validation; and 0.657 [0.624–0.690] in external validation). High ALNCR (≥ 3.21) was associated with longer OS than low ALNCR (median OS not reached vs. 22.3 months; log‐rank *p* < 0.001). After multivariable adjustment, high ALNCR remained associated with lower mortality (HR 0.56 [0.51–0.61], 0.50 [0.43–0.58] and 0.53 [0.42–0.67] across the three cohorts; all *p* < 0.001). Adding ALNCR to TNM improved prediction over TNM alone (ΔC‐index +0.021; NRI 0.067, *p* = 0.010; IDI 0.004, *p* < 0.001) and stratified patients into four risk groups (*p* < 0.0001), with consistent associations across major cancer types and clinical strata.

**Conclusions:**

ALNCR is a simple inflammation‐based prognostic index that outperforms conventional inflammatory markers for OS prediction. Integrating ALNCR with TNM stage improves risk stratification and prognostic accuracy, supporting clinical implementation via a nomogram and web‐based calculator.

## 1 Introduction

The incidence and mortality of malignant tumours continue to rise globally, posing a major public health challenge [1]. According to the World Health Organization, the number of new cancer cases worldwide is projected to reach 28.4 million by 2040, a 47% increase compared to 2020 [2]. Accurate prediction of cancer progression and patient prognosis has become a critical issue. Advances in multiomics technologies have enabled systematic investigations into tumour characteristics and progression. By analysing genomic alterations and protein expression, these methods provide valuable insights into cancer staging, treatment and prognosis [3, 4]. However, their reliance on invasive procedures (e.g., biopsies) or expensive molecular tests limits their accessibility and widespread clinical application.

Systemic inflammation plays a pivotal role in tumour initiation, progression, metastasis and prognosis. It reflects immune responses and cytokine networks within the tumour microenvironment and is closely associated with patient survival [5]. Biomarkers such as albumin, lymphocytes, neutrophils, platelets and C‐reactive protein (CRP) have been widely used to assess systemic inflammation [6, 7, 8, 9]. In recent years, several inflammation‐based prognostic indices have been derived from these markers, including the neutrophil‐to‐lymphocyte ratio (NLR) [10], platelet‐to‐lymphocyte ratio (PLR) [11], CRP‐to‐albumin ratio (CAR) [11], systemic immune‐inflammation index (SII) [12] and prognostic nutritional index (PNI) [13]. Although these indices have shown predictive potential in certain cancer types, their applicability across diverse populations and cancer subtypes remains limited, requiring further validation.

To enhance the prognostic value of systemic inflammation, this study proposes a novel inflammatory burden index, ALNCR, based on the combination of albumin, lymphocytes, neutrophils and CRP. ALNCR is defined as (albumin [g/dL] × lymphocyte [/μL]) / (neutrophil [/μL] × C‐reactive protein [mg/dL]). This index was designed based on the biological rationale that albumin and lymphocytes reflect nutritional and immune status [14, 15], whereas neutrophils and CRP represent systemic inflammatory burden [16, 17]. By integrating these components, ALNCR provides a more comprehensive assessment of the interplay between nutrition, immune response and inflammation in cancer prognosis. Furthermore, we integrated ALNCR with the widely used TNM staging system to create the ALNCR.TNM model, recognizing that inflammation significantly contributes to tumour progression beyond anatomical staging alone. Through comparative analyses, we evaluated its potential for clinical application, offering new perspectives for personalized treatment and prognosis assessment.

## 2 Method

### 2.1 Study Design

This retrospective cohort study utilized data from the INSCOC (Investigation on Nutrition Status and its Clinical Outcome of Common Cancers) cancer and patient nutrition project, a multicentre database that collected clinical and nutritional data from cancer patients across hospitals in China between 2013 and 2021. Cancer patients with available clinical and laboratory data were included for analysis. The study was conducted in two phases: First, a training cohort and an internal validation cohort were derived from the INSCOC database, and second, an external validation cohort was obtained from Fujian Cancer Hospital to assess the generalizability of the proposed prognostic model. Specifically, the INSCOC cohort was randomly split in a prespecified 70/30 ratio into a training set (model development) and an internal validation set (hold‐out evaluation). There is no universally mandated guideline for an exact split ratio; we used the commonly adopted 70/30 rule‐of‐thumb to retain a large development sample while reserving a sufficiently sized independent test set for internal evaluation and further assessed transportability in an independent external cohort [18] The inclusion criteria were as follows: (1) histologically confirmed diagnosis of malignant tumour, (2) age ≥ 18 years and (3) availability of complete pretreatment laboratory test results, including albumin, lymphocyte count, neutrophil count and C‐reactive protein (CRP). Patients were excluded if they had (1) missing key clinical data, (2) concurrent autoimmune diseases or chronic inflammatory conditions or (3) received immunosuppressive therapy prior to baseline assessment. This study received ethical approval from the institutional review boards of the participating medical centres. Written informed consent was obtained from all patients before participation.

### 2.2 Patient Characteristics

Baseline clinical characteristics were extracted from electronic health records and included demographic information (age, sex and body mass index [BMI]), tumour‐related variables (TNM stage and tumour type), lifestyle factors (smoking and alcohol consumption), comorbidities (hypertension, diabetes and coronary artery disease) and treatment history (surgery, chemotherapy and radiotherapy). TNM staging was classified according to the eighth edition of the American Joint Committee on Cancer (AJCC) TNM staging system. BMI was calculated as weight (kg) divided by height squared (m^2^) and categorized based on the Chinese population criteria: underweight (< 18.5 kg/m^2^), normal weight (18.5–23.9 kg/m^2^), overweight (24.0–27.9 kg/m^2^) and obese (≥ 28 kg/m^2^). Venous blood samples were collected by trained medical staff after at least 8 h of fasting and within 48 h prior to treatment initiation. Laboratory indicators included albumin, lymphocytes, neutrophils, CRP and other routine biochemical markers. Data were recorded in a standardized format to ensure consistency across study sites.

### 2.3 Follow‐Up and Endpoint Assessment

Patient follow‐up was conducted through annual hospital visits and telephone interviews, starting from the date of initial cancer diagnosis. Clinical data were regularly updated using electronic medical records and direct patient communication to ensure data accuracy. The primary endpoint of this study was overall survival (OS), defined as the time from cancer diagnosis to death from any cause or the last recorded follow‐up date.

### 2.4 Definition and Calculation of Prognostic Indices

To comprehensively assess the inflammatory burden and nutritional status of cancer patients, several inflammatory and prognostic indices were calculated based on pretreatment laboratory data. These indices included neutrophil‐to‐lymphocyte ratio (NLR), platelet‐to‐lymphocyte ratio (PLR), C‐reactive protein‐to‐albumin ratio (CAR), systemic immune‐inflammation index (SII), prognostic nutritional index (PNI) and ALNCR (Figure S1). The calculation methods for these prognostic indices are provided in Table S1.

### 2.5 Statistical Analysis

Continuous variables were presented as mean ± standard deviation (SD) or median with interquartile range (IQR), depending on the distribution. Normality was assessed using the Shapiro–Wilk test. Comparisons between groups were performed using the Student's *t* test for normally distributed variables and the Mann–Whitney *U* test for nonnormally distributed variables. Categorical variables were expressed as frequencies (percentages) and analysed using the *χ* ^2^ test or Fisher's exact test, as appropriate. The optimal cut‐off value for ALNCR was determined using maximally selected rank statistics. The cut‐off was derived in the training cohort and then applied unchanged in the internal validation and external validation cohorts to evaluate reproducibility without reoptimization. To evaluate the discriminative ability of ALNCR and other inflammatory indices, we calculated the C‐index and time‐dependent receiver operating characteristic (ROC) curves with the area under the curve (AUC) for overall survival (OS). The prognostic impact of ALNCR was further assessed using Kaplan–Meier survival analysis, and differences between survival curves were compared using the log‐rank test. Univariate and multivariate Cox proportional hazards regression analyses were conducted to identify independent prognostic factors for OS. The multivariate Cox model was adjusted for potential confounders, including tumour stage, BMI, diabetes, hypertension, coronary artery disease, smoking, alcohol consumption, surgery, chemotherapy and radiotherapy. Restricted cubic spline (RCS) models were used to evaluate the nonlinear association between ALNCR and survival risk. To further improve risk stratification, we developed the ALNCR.TNM combined model, which was compared with ALNCR and TNM alone using net reclassification improvement (NRI) and integrated discrimination improvement (IDI) analyses. Statistical significance was set at *p* < 0.05 (two‐sided tests). The 70/30 split was prespecified and implemented by random allocation (with a fixed random seed) to ensure reproducibility [19]. All analyses were conducted using R software (version 4.2.1).

## 3 Result

### 3.1 Study Participants and Characteristics

A total of 6374 cancer patients were included in this study. These patients were randomly divided into two groups in a 7:3 ratio, resulting in an internal training cohort (4464 patients) and an internal validation cohort (1910 patients). Additionally, an independent external validation cohort of 759 patients was included from Fujian Cancer Hospital to further validate the findings (Figure S2). Baseline characteristics are summarized in Table 1. The male proportions were 61.0%, 58.8% and 68.0% for the training, internal validation and external validation cohorts, respectively. The median ages were 60.0, 60.0 and 58.0 years for the three cohorts. Smoking and drinking rates were comparable across all cohorts. Notably, chemotherapy was more frequent in the external validation cohort (76.9%), indicating a higher treatment intensity. The most common tumour types were lung cancer (31.8%, 32.2% and 12.4%), colorectal cancer (19.3%, 20.9% and 30.2%) and gastric cancer (15.5%, 15.1% and 29.5%). The external validation cohort had a higher proportion of advanced‐stage tumours, with 64.8% in stage IV compared to 45.4% and 45.8% in the training and internal validation cohorts, respectively, reflecting a greater disease burden. Additionally, inflammatory and nutritional markers varied, with the median ALNCR being lower in the external validation cohort (2.87 vs. 3.47 and 3.34), along with differences in albumin and CRP levels.

**TABLE 1:** Comparison of demographic and clinicopathological characteristics across the internal training, internal validation, and external validation cohorts.

Variable	Internal training cohort (*n* = 4464)	Internal validation cohort (*n* = 1910)	External validation cohort (*n* = 759)
Gender, male (%)	2722.00 (61.00)	1123.00 (58.8)	516 (68.0)
Age, years, median (IQR)	60.00 (52.00, 67.00)	60.00 (53.00, 67.00)	58.00 (49.00, 66.00)
Smoking, yes, *n* (%)	2114 (47.4)	899 (47.1)	338 (44.5)
Drinking, yes, *n* (%)	1032 (23.1)	426 (22.3)	151 (19.9)
Chemotherapy, yes, *n* (%)	2584 (57.9)	1088 (57.0)	584 (76.9)
Radiotherapy, yes, *n* (%)	258 (5.8)	115 (6.0)	34 (4.5)
Diabetes, yes, *n* (%)	479 (10.7)	182 (9.5)	68 (9.0)
Hypertension, yes, *n* (%)	894 (20.0)	393 (20.6)	143 (18.8)
Coronary disease, yes, *n* (%)	222 (5.0)	94 (4.9)	21 (2.8)
Family history, yes, *n* (%)	742 (16.6)	318 (16.6)	55 (7.2)
Surgery, yes, *n* (%)	804 (18.0)	373 (19.5)	38 (5.0)
Cachexia, yes, *n* (%)	1525 (34.2)	650 (34.0)	258 (34.0)
BMI group (%)			
Normal weight	529 (11.9)	222 (11.6)	108 (14.2)
Overweight	2457 (55.0)	1057 (55.3)	446 (58.8)
Obesity	1478 (33.1)	631 (33.0)	205 (27.0)
Tumour stage, *n* (%)			
I	422 (9.5)	184 (9.6)	34 (4.5)
II	847 (19.0)	343 (18.0)	84 (11.1)
III	1168 (26.2)	509 (26.6)	149 (19.6)
IV	2027 (45.4)	874 (45.8)	492 (64.8)
Tumour type, *n* (%)			
Lung cancer	1419 (31.8)	615 (32.2)	94 (12.4)
Oesophageal cancer	273 (6.1)	106 (5.5)	54 (7.1)
Gastric cancer	693 (15.5)	288 (15.1)	224 (29.5)
Liver cancer	187 (4.2)	81 (4.2)	16 (2.1)
Pancreatic cancer	112 (2.5)	39 (2.0)	16 (2.1)
Colorectal cancer	861 (19.3)	399 (20.9)	229 (30.2)
Urinary system tumour	153 (3.4)	46 (2.4)	0 (0.0)
Nasopharyngeal carcinoma	124 (2.8)	52 (2.7)	15 (2.0)
Breast cancer	343 (7.7)	160 (8.4)	36 (4.7)
Gynaecological tumour	163 (3.7)	82 (4.3)	6 (0.8)
Others	136 (3.0)	42 (2.2)	69 (9.1)
Albumin, g/L, median (IQR)	39.30 (35.60, 42.40)	39.30 (35.50, 42.40)	38.30 (34.60, 41.20)
CRP, mg/L, median (IQR)	4.22 (2.41, 19.51)	4.10 (2.44, 18.50)	4.30 (3.20, 20.90)
Hb, g/L, median (IQR)	125.00 (110.00, 138.00)	125.00 (110.00, 138.00)	124.00 (110.00, 136.00)
WBC, 10^9^/L, median (IQR)	6.00 (4.70, 7.81)	6.00 (4.65, 7.82)	6.20 (4.90, 8.20)
Neutrophils, 10^9^/L, median (IQR)	3.79 (2.62, 5.40)	3.72 (2.61, 5.47)	4.00 (2.90, 5.70)
Lymphocyte, 10^9^/L, median (IQR)	1.44 (1.03, 1.88)	1.43 (1.03, 1.85)	1.50 (1.10, 1.90)
RBC, 10^12^/L, median (IQR)	4.23 (3.78, 4.63)	4.22 (3.79, 4.62)	4.19 (3.79, 4.62)
PLT, 10^9^/L, median (IQR)	218.00 (167.00, 279.00)	223.00 (170.00, 286.00)	242.00 (190.00, 301.00)
PGSGA	3.00 (0.00, 7.00)	3.00 (1.00, 7.00)	3.00 (1.00, 7.00)
LOS, day	10.00 (6.00, 16.00)	10.00 (6.00, 16.75)	10.00 (8.00, 15.00)
NLR	2.58 (1.71, 4.20)	2.59 (1.71, 4.33)	2.64 (1.77, 4.06)
PLR	151.11 (105.61, 222.67)	155.55 (111.50, 222.03)	154.00 (115.00, 222.45)
CAR	0.11 (0.06, 0.52)	0.11 (0.06, 0.52)	0.12 (0.08, 0.55)
SII	571.21 (327.79, 992.22)	569.08 (335.27, 1018.95)	630.00 (371.72, 1032.96)
PNI	46.80 (42.20, 50.90)	46.80 (41.91, 50.59)	383.01 (346.00, 412.01)
ALNCR	3.47 (0.55, 10.17)	3.34 (0.58, 10.27)	2.87 (0.47, 6.17)

Abbreviations: ALNCR, albumin × lymphocyte to neutrophil × C‐reactive protein ratio; BMI, body mass index; CAR, C‐reactive protein‐to‐albumin ratio; CRP, C‐reactive protein; Hb, haemoglobin; LOS, length of hospital stay; NLR, neutrophil‐to‐lymphocyte ratio; PGSGA, patient‐generated subjective global assessment; PLT, platelet; PNI, prognostic nutritional index; RBC, red blood cell; SII, systemic immune‐inflammation index; WBC, white blood cell.

### 3.2 Prognostic Superiority of ALNCR Index in Cancer Patients

Systemic inflammation exacerbation is typically characterized by decreased albumin and lymphocytes, alongside increased neutrophils, platelets and C‐reactive protein (CRP). Based on these inflammatory markers, we systematically compared multiple prognostic indices to identify the optimal predictor for cancer prognosis. ALNCR was evaluated against classical inflammation‐based indices, including NLR, PLR, CAR, SII and PNI, using C‐index and time‐dependent AUC metrics across three cohorts. Calculation formulas for all indices are provided in Table S2. Results demonstrated that in both the internal training and validation cohorts, ALNCR achieved C‐indices of 0.645 (95% CI: 0.631–0.658) and 0.666 (95% CI: 0.645–0.686), respectively, outperforming all other indices. In the external validation cohort, ALNCR remained the highest‐ranking predictor with a C‐index of 0.657 (95% CI: 0.624–0.690), confirming its stability and reproducibility across independent populations (Table S2). Similarly, time‐dependent AUC analysis further validated ALNCR's predictive superiority. Compared to other indices, ALNCR exhibited consistently higher AUC values across all time points in the internal training (Figure S3A), internal validation (Figure S3B) and external validation cohorts (Figure S3C). These findings indicate that ALNCR possesses robust generalizability and reliability, making it a promising prognostic tool for cancer patients.

### 3.3 Association Between ALNCR and Survival Outcomes in Cancer

**FIGURE 1:**
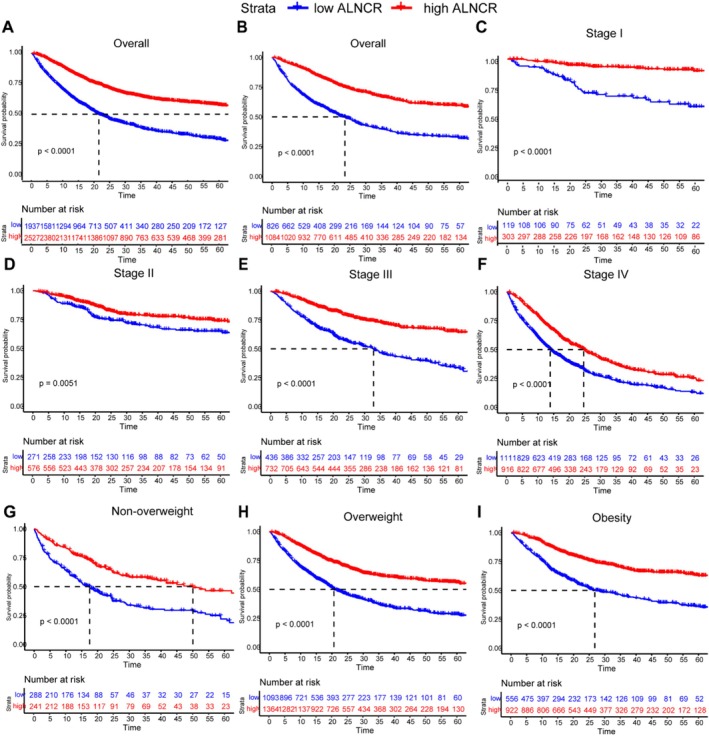
Kaplan–Meier survival curves stratified by ALNCR in overall and subgroup populations. Kaplan–Meier survival analysis of overall survival (OS) according to ALNCR levels across different patient subgroups. (A) Total population in the internal training cohort. (B) Total population in the internal validation cohort. (C–F) PATIENTS with Tumour Stages I–IV. (G–I) Patients stratified by body mass index (BMI): nonoverweight, overweight and obese groups. *p* values were calculated using the log‐rank test.

The optimal cut‐off value for ALNCR was 3.21 in the training cohort (Figure S4) and was applied to all validation analyses. The corresponding optimal cut‐off in the external cohort was similar (3.18). Using this threshold, patients were categorized into high ALNCR (≥ 3.21) and low ALNCR (< 3.21) groups. Kaplan–Meier survival curves demonstrated that patients in the high ALNCR group exhibited significantly better survival outcomes across all cohorts, including the internal and external validation cohorts (Figure 1A,B). When stratified by TNM stage, high ALNCR remained associated with improved survival outcomes in Stages I–IV patients (Figure 1C–F), suggesting its prognostic robustness across different cancer stages. Similarly, in analyses stratified by BMI categories (normal weight, overweight and obese), high ALNCR consistently predicted better survival in each BMI subgroup (Figure 1G–I).

Subgroup analyses by cancer type further validated the prognostic utility of ALNCR. Patients with high ALNCR had significantly better survival in lung cancer, oesophageal cancer, gastric cancer, liver cancer, pancreatic cancer, colorectal cancer, urological tumours, nasopharyngeal cancer, breast cancer and gynaecological cancers (Figure S5A–L). These findings underscore the broad applicability of ALNCR in predicting prognosis across diverse patient populations, cancer stages and tumour types.

### 3.4 Independent Prognostic Value of ALNCR in Subgroups

**FIGURE 2:**
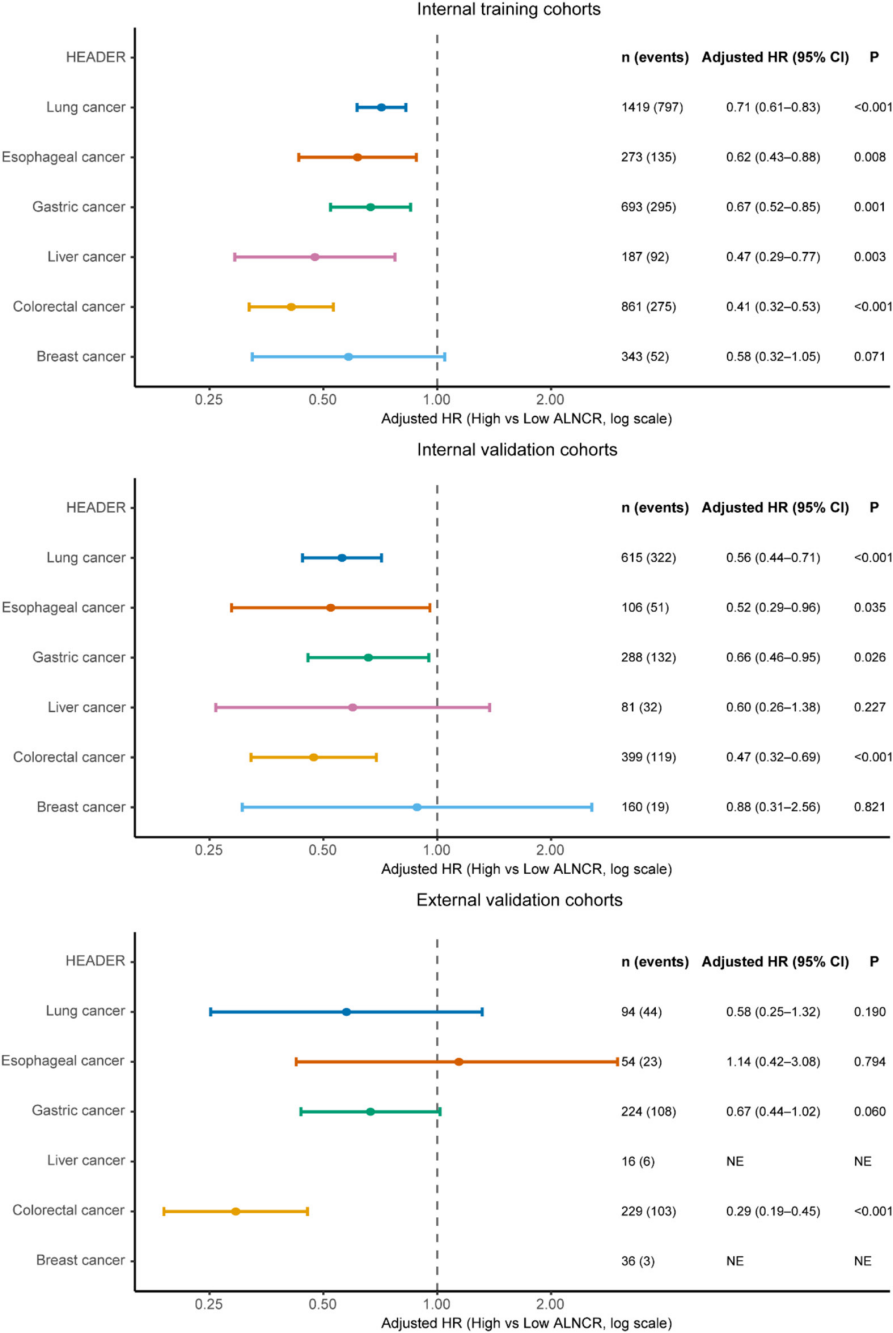
Cancer type–stratified prognostic value of ALNCR across cohorts. Forest plots show adjusted hazard ratios (HRs) and 95% confidence intervals (CIs) for overall survival comparing high versus low ALNCR (cut‐off = 3.21) within the six most prevalent cancer types (lung, oesophageal, gastric, liver, colorectal and breast cancer) in the internal training cohort (top), internal validation cohort (middle) and external validation cohort (bottom). Estimates were obtained from cancer type–stratified multivariable Cox proportional hazards models adjusted for the same covariates as in Table 2. Points indicate HRs and horizontal lines indicate 95% CIs on a log scale; the dashed vertical line denotes HR = 1. Numbers to the right indicate total sample size and number of events for each cancer type. NE, not estimable, indicates insufficient events or model nonconvergence in that subgroup.

To evaluate the independent prognostic value of ALNCR, we conducted univariate and multivariate Cox regression analyses in the internal training cohort, internal validation cohort and external validation cohort (Table 2). High ALNCR values were consistently associated with a significantly lower risk of mortality compared to low ALNCR across all cohorts (HR = 0.59, 95% CI: 0.53–0.65, *p* < 0.001 in the training cohort). This protective effect remained robust in the internal and external validation cohorts, confirming the reproducibility of ALNCR's prognostic value. After adjusting for confounding factors, including age, sex, BMI and tumour stage, high ALNCR values continued to demonstrate significant protective effects in all three cohorts. To assess whether the prognostic value of ALNCR was driven by a single laboratory parameter, we conducted a multivariable Cox model including the four score components (albumin, lymphocyte count, neutrophil count and CRP) simultaneously and adjusted for the same covariates as in Table 2. Each component was log‐transformed and standardized (per 1‐SD increase). The directions of association were consistent across cohorts (higher CRP/neutrophils associated with worse OS; higher albumin/lymphocytes associated with better OS), supporting that ALNCR captures joint inflammatory and nutritional/immune information (Table S3). Sensitivity analyses validated the stability of these findings, showing consistent results even after excluding patients with short‐term mortality or those with coronary heart disease (Table S4). Nonlinear survival risk curves, visualized using restricted cubic splines, demonstrated a sharp reduction in mortality risk with increasing ALNCR values, with the risk plateauing at higher levels (Figure S6A–C). These results were consistent across different adjustment models (Figure S6D–F). Subgroup analyses revealed that ALNCR provided significant prognostic value in diverse patient populations, including those stratified by age, sex, tumour stage and treatment history (Figure S7). For instance, the protective effect of high ALNCR was particularly pronounced in younger patients (< 65 years, HR = 0.64, 95% CI: 0.57–0.72) and early‐stage cancer patients (Stage I, HR = 0.28, 95% CI: 0.16–0.51). To enhance individual cancer‐type relevance, we further performed cancer type–stratified multivariable Cox models (high vs. low ALNCR; cut‐off = 3.21) in the six most prevalent malignancies and summarized the adjusted hazard ratios across the internal training, internal validation and external validation cohorts in Figure 2. In the internal training and internal validation cohorts, the association between high ALNCR and improved overall survival was generally consistent across cancer types, whereas in the external validation cohort, several cancer‐type estimates were nonestimable due to limited sample size/events.

**TABLE 2:** Univariate and multivariate survival analysis of clinicopathological characteristics.

Variable	Level	Model	Training cohort HR (95% CI)	*p* value	Validation cohort HR (95% CI)	*p* value	External cohort HR (95% CI)	*p* value
Age ≥ 65		Univ. COX	1.02 (1.01, 1.02)	< 0.001	1.02 (1.01, 1.03)	< 0.001	1.00 (0.99, 1.01)	0.779
		Multiv. COX	1.01 (1.01, 1.02)	< 0.001	1.01 (1.00, 1.02)	0.008	1.00 (0.99, 1.01)	0.563
Gender (male)		Univ. COX	0.69 (0.63, 0.76)	< 0.001	0.69 (0.60, 0.80)	< 0.001	0.77 (0.61, 0.98)	0.033
		Multiv. COX	0.77 (0.70, 0.84)	< 0.001	0.75 (0.65, 0.87)	< 0.001	0.87 (0.64, 1.18)	0.033
Family history (yes)		Univ. COX	0.93 (0.82, 1.05)	0.250	0.80 (0.66, 0.98)	0.030	0.89 (0.58, 1.35)	0.570
		Multiv. COX	0.99 (0.87, 1.12)	0.830	0.85 (0.70, 1.04)	0.122	0.88 (0.78, 0.99)	0.469
Smoking (yes)		Univ. COX	1.40 (1.28, 1.53)	< 0.001	1.35 (1.17, 1.55)	< 0.001	1.26 (1.02, 1.57)	0.036
		Multiv. COX	1.15 (1.03, 1.29)	0.010	1.19 (1.00, 1.41)	0.049	1.02 (0.78, 1.34)	0.881
Drinking (yes)		Univ. COX	1.15 (1.04, 1.28)	0.008	1.24 (1.06, 1.46)	0.008	1.09 (0.83, 1.43)	0.523
		Multiv. COX	0.97 (0.86, 1.08)	0.547	1.14 (0.95, 1.36)	0.157	1.17 (0.88, 1.56).	0.278
Tumour stage	I	Univ. COX.	Ref.		Ref.		Ref.	
	II	Univ. COX	0.36 (0.31,0.42)	< 0.001	0.34 (0.43, 0.43)	< 0.001	0.33 (0.20, 0.53)	< 0.001
	II	Multiv. COX	1.68 (1.26, 2.25)	< 0.001	1.68 (1.26, 2.25)	< 0.001	1.68 (1.26, 2.25)	< 0.001
	III	Univ. COX	0.67 (0.60, 0.75)	< 0.001	0.84 (0.71, 0.99)	0.04	0.37 (0.26, 0.52)	< 0.001
	III	Multiv. COX	2.91 (2.22, 3.82)	< 0.001	2.91 (2.22, 3.82)	< 0.001	2.91 (2.22, 3.81)	< 0.001
	IV	Univ. COX	3.77 (3.42, 4.15)	< 0.001	3.17 (2.73, 3.68)	< 0.001	4.42 (3.31, 5.91)	< 0.001
	IV	Multiv. COX	7.22 (5.56, 9.39)	< 0.001	7.22 (5.55, 9.39)	< 0.001	7.22 (5.55, 0.39)	< 0.001
Surgery (yes)		Univ. COX	1.92 (1.67, 2.19)	< 0.001	1.78 (1.45, 2.17)	< 0.001	0.36 (0.17, 0.76)	0.007
		Multiv. COX	1.23 (1.06, 1.42)	0.005	1.18 (0.96, 1.47)	0.121	0.78 (0.36, 1.67)	0.53
Chemotherapy (yes)		Univ. COX	0.83 (0.76, 0.92)	< 0.001	0.96 (0.83, 1.01)	0.542	1.07 (0.82, 1.41)	0.602
		Multiv. COX	0.98 (0.0.89, 1.08)	0.705	1.04 (0.90, 1.21)	0.565	1.05 (0.80, 1.38)	0.713
Radiotherapy (yes)		Univ. COX	1.30 (1.05, 1.61)	0.02	1.02 (0.76, 1.37)	0.89	0.53 (0.26, 1.07)	0.08
		Multiv. COX	1.49 (1.20, 1.85)	< 0.001	1.25 (0.93, 1.68)	0.138	0.42 (0.21, 0.85)	0.016
Diabetes (yes)		Univ. COX	1.25 (1.09, 1.43)	0.001	1.24 (0.98, 1.55)	0.07	1.21 (0.08, 1.77)	0.315
		Multiv. COX	1.18 (1.02, 1.35)	0.022	1.24 (0.98, 1.56)	0.07	1.22 (0.83, 1.79)	0.308
Hypertension (yes)		Univ. COX	1.19 (1.06, 1.32)	0.002	1.12 (0.94, 1.33)	0.22	1.02 (0.76, 1.35)	0.87
		Multiv. COX	1.12 (1.00, 1.26)	0.052	1.10 (0.91, 1.32)	0.314	1.13 (0.84, 1.52)	0.417
Coronary disease (yes)		Univ. COX	0.91 (0.74, 1.06)	0.388	1.13 (0.82, 1.56)	0.460	0.83 (0.42, 1.61)	0.575
		Multiv. COX	0.85 (0.69, 1.06)	0.144	1.10 (0.79, 1.53)	0.577	1.22 (0.56, 2.21)	0.739
Cachexia (yes)		Univ. COX	1.57 (1.43, 1.72)	< 0.001	1.56 (1.35, 1.81)	< 0.001	1.32 (1.06, 1.65)	0.013
		Multiv. COX	1.33 (1.20, 1.47)	< 0.001	1.31 (1.12, 1.53)	< 0.001	1.36 (1.07, 1.72)	0.011
ALNCR (> 3.21)		Univ. COX	0.43 (0.39, 0.47)	< 0.001	0.40 (0.35, 0.46)	< 0.001	0.38 (0.30, 0.49)	< 0.001
		Multiv. COX	0.56 (0.51, 0.61)	< 0.001	0.50 (0.43, 0.58)	< 0.001	0.53 (0.42, 0.67)	< 0.001

*Note:* Univ. COX indicates univariable Cox proportional hazards regression (unadjusted). Multiv. COX indicates multivariable Cox proportional hazards regression adjusted for age, sex, BMI, tumour type, tumour stage, surgery, chemotherapy, radiotherapy, smoking, drinking, family history, diabetes, hypertension, coronary heart disease, and cachexia.

Abbreviations: ALNCR, albumin–lymphocyte–neutrophil–CRP ratio; CI, confidence interval; HR, hazard ratio.

### 3.5 Distribution of ALNCR Across TNM Stages and Cancer Types

We further analysed the distribution of ALNCR across different TNM stages and cancer types. Figure S8 shows that ALNCR decreases as TNM stages progress, suggesting that higher ALNCR values are associated with better prognoses in early stages, whereas lower values in advanced stages reflect greater disease aggressiveness and systemic inflammation. Figure S9 illustrates the distribution of ALNCR among cancer types. Patients with lung, gastric and pancreatic cancers had relatively higher ALNCR values, whereas those with colorectal and breast cancers had lower levels. These differences likely reflect tumour‐specific pathophysiological characteristics and their distinct impacts on systemic inflammation and immune responses.

### 3.6 Performance of the ALNCR.TNM Combined Model

**FIGURE 3:**
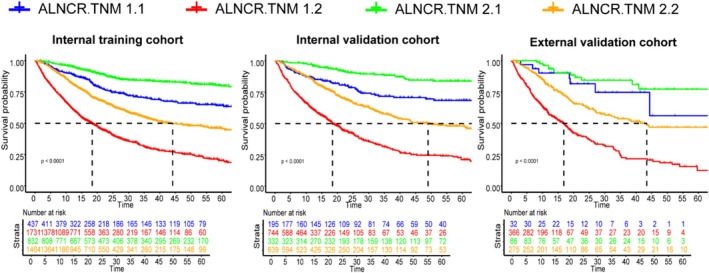
Kaplan–Meier survival curves for ALNCR.TNM subgroups. Kaplan–Meier curves showing overall survival across combined ALNCR and TNM stage subgroups in the internal training, internal validation and external validation cohorts.

Whereas ALNCR independently predicts prognosis, TNM staging, a classic clinical tool based on anatomical features, cannot reflect the role of systemic inflammation in tumour progression. To address this, we developed the ALNCR.TNM model by combining ALNCR (categorized as high ≥ 3.21 and low < 3.21) and TNM staging (early: I/II; late: III/IV). Patients were divided into four subgroups: 1.1 (low ALNCR + early TNM), 1.2 (low ALNCR + late TNM), 2.1 (high ALNCR + early TNM) and 2.2 (high ALNCR + late TNM). The combined model outperformed ALNCR or TNM staging alone in C‐index (Table S5) and AUC (Figure S10). Kaplan–Meier survival curves demonstrated that the ALNCR.TNM model effectively stratified survival risk (Figure 3). Group 2.1 showed the highest survival rates, whereas Group 1.2 had the lowest (*p* < 0.0001).

For reclassification, the ALNCR.TNM model showed a significant improvement over TNM (NRI = 0.067, 95% CI: 0.016–0.118, *p* = 0.010) but not over ALNCR alone (NRI = 0.010, 95% CI: −0.023–0.044, *p* = 0.542). Incremental discrimination analysis showed a significant improvement compared to TNM (IDI = 0.004, 95% CI: 0.002–0.005, *p* < 0.001) but not over ALNCR (IDI = 0.000, 95% CI: 0.000–0.001, *p* = 0.200) (Figure S11). These results suggest that the ALNCR.TNM model enhances prognostic performance over TNM staging.

### 3.7 Clinical Utility and Validation of the ALNCR‐TNM Model

**FIGURE 4:**
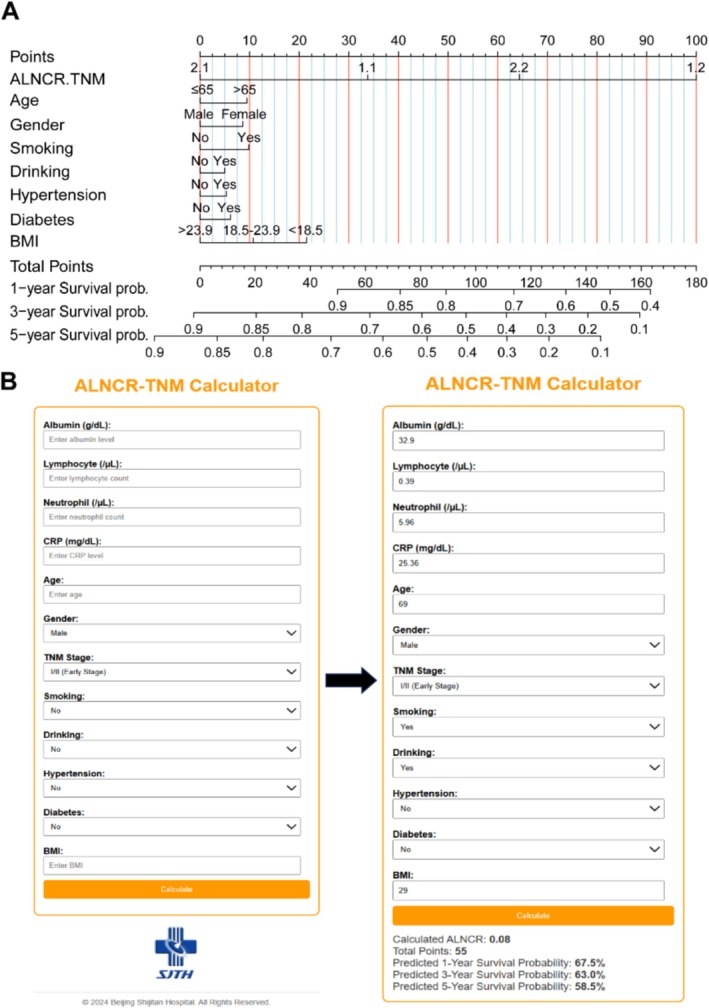
Nomogram and calculator for predicting overall survival of cancer patients. (A) Nomogram integrating ALNCR and TNM stage for individualized prediction of 1‐, 3‐ and 5‐year overall survival. (B) ALNCR.TNM calculator providing the predicted survival probability for a given patient based on the nomogram model.

To further validate the clinical utility of the ALNCR.TNM combined model, we developed a nomogram based on the Cox proportional hazards regression model (Figure 4A). This nomogram predicts the 1‐, 3‐ and 5‐year survival probabilities for cancer patients by integrating ALNCR.TNM subgroups and other clinical variables (e.g., BMI, smoking, drinking, hypertension and diabetes). It offers clinicians an accessible and visual tool for individualized risk assessment. Additionally, we created a web‐based calculator (Figure 4B) that allows clinicians to easily calculate a patient's ALNCR value and predict survival rates by entering biological indicators (albumin, lymphocytes, neutrophils and C‐reactive protein). The nomogram's predictive performance for 1‐, 3‐ and 5‐year survival was confirmed through calibration curves (Figure S12A–C).

Nomograms based on the ALNCR.TNM model were also developed for specific cancer types, including lung cancer, gastric cancer, oesophageal cancer, colorectal cancer, liver cancer and breast cancer (Figure S13A–F). These nomograms combine ALNCR.TNM grouping with clinical variables, such as smoking, drinking, hypertension, diabetes and BMI categories, to predict survival probabilities for each cancer type. They provide clinicians with an efficient tool for individualized survival prediction and risk stratification, demonstrating the wide applicability of the ALNCR.TNM model in clinical practice.

## 4 Discussion

In this study, we developed and validated a novel inflammatory burden index, ALNCR, derived from a combination of albumin, lymphocytes, neutrophils and C‐reactive protein (CRP) to predict cancer prognosis. Furthermore, we integrated ALNCR with the widely used TNM staging system to create the ALNCR.TNM model, which demonstrated superior prognostic performance in comparison to ALNCR or TNM staging alone.

Our study shows that ALNCR, a simple and effective inflammation‐based index, outperforms other inflammation‐related markers, including NLR, PLR, CAR, SII and PNI, in predicting cancer prognosis. Compared to these conventional indices, ALNCR integrates systemic inflammation with host condition, combining markers reflecting the inflammatory response (CRP, neutrophils) and markers related to immune cell balance and hepatic synthetic/negative acute‐phase response (lymphocytes, albumin). Importantly, serum albumin should not be interpreted as a standalone marker of nutritional status, as it is a negative acute‐phase reactant and is strongly influenced by inflammation and disease severity [20]. Consistently, when we modelled the four components simultaneously (log‐transformed and standardized), their directions aligned with the biological interpretation (higher CRP/neutrophils associated with worse OS; higher albumin/lymphocytes associated with better OS), supporting that ALNCR captures joint information rather than being driven by albumin alone (Table S3). The consistent superiority of ALNCR across different cohorts suggests that systemic inflammation and nutritional status are fundamentally linked to cancer progression and patient survival. Albumin serves as a vital nutritional reserve and plays a critical role in key physiological processes, including immune regulation and fluid homeostasis [21, 22]. Cancer progression is often associated with hypoalbuminemia, which can result from impaired hepatic synthesis, insufficient nutritional intake and metabolic dysregulation in cancer patients [22, 23, 24]. In the ALNCR formula, albumin and CRP serve as indicators of systemic inflammation and metabolic response, whereas lymphocytes and neutrophils reflect host immune competence and tumour‐associated inflammation. By integrating these factors, ALNCR offers a more comprehensive evaluation of the inflammatory burden in cancer patients. These findings align with emerging evidence highlighting the critical role of systemic inflammation in modulating cancer progression, immune evasion and response to therapy.

The role of systemic inflammation in cancer progression has been well established. Inflammation in the tumour microenvironment is closely linked to immune responses, cytokine release and tumour metastasis, all of which affect patient survival [25, 26]. Our findings align with previous studies showing that inflammatory markers such as CRP and albumin are significant predictors of cancer outcomes. However, our study provides a novel contribution by combining these markers into the ALNCR index, which offers a more comprehensive and accurate assessment of the systemic inflammatory burden. The ALNCR index was not only predictive in the overall population but also demonstrated prognostic power across various subgroups. Our analysis by TNM stage revealed that ALNCR is consistently associated with improved survival, even in advanced‐stage cancer patients. This suggests that ALNCR may complement TNM staging by adding an inflammatory dimension to the conventional anatomical classification. Furthermore, subgroup analyses by BMI and cancer type demonstrated that ALNCR holds prognostic value across diverse patient populations, indicating its broad applicability. One of the key strengths of our study is the development of the ALNCR.TNM combined model, which incorporates both systemic inflammation and tumour stage. This model provides a more refined stratification of patient prognosis, particularly when compared to TNM staging alone. The ALNCR.TNM model achieved superior C‐index values and showed better discriminatory power in Kaplan–Meier survival analysis. Patients classified into the ALNCR.TNM 2.1 subgroup (high ALNCR and early TNM stage) had the highest survival probability, whereas those in the ALNCR.TNM 1.2 subgroup (low ALNCR and late TNM stage) had the poorest prognosis. These findings suggest that the ALNCR.TNM model provides a more nuanced and individualized approach to predicting cancer outcomes. Our reclassification analysis further supports the clinical utility of the ALNCR.TNM model. The model significantly improved the predictive accuracy over TNM staging alone, as evidenced by the positive NRI and IDI values. However, the improvement over ALNCR alone was modest, indicating that although ALNCR is a powerful predictor on its own, its integration with TNM staging offers additional prognostic value. This is consistent with the understanding that both tumour biology (TNM staging) and systemic factors (inflammation) are crucial for cancer prognosis.

The clinical utility of the ALNCR.TNM model was further validated through the development of a nomogram, which provides a simple, visual representation of individual survival probabilities. The nomogram allows clinicians to incorporate patient‐specific variables, including ALNCR.TNM subgroups and other clinical factors, to predict 1‐, 3‐ and 5‐year survival rates. This tool can facilitate personalized treatment decisions, especially in complex cancer cases where treatment options and prognostic evaluation are critical. Additionally, we created a web‐based calculator that automates the process of calculating ALNCR and predicting survival probabilities. This digital tool is designed for easy use in clinical practice, enabling rapid and accurate risk assessment without the need for extensive computational expertise. The positive validation of the nomogram and calculator, as well as the calibration curves, confirms that these tools are reliable and effective in predicting cancer survival.

Given that ALNCR is derived from routinely available pre‐treatment laboratory tests, it can be implemented as an adjunct to TNM staging for risk stratification at diagnosis. In practice, ALNCR (or ALNCR.TNM) may help identify patients with an unfavourable inflammation–host condition profile within the same anatomical stage, supporting closer surveillance and earlier supportive assessment (e.g., evaluation of infection/inflammatory complications, treatment tolerance and supportive care needs) and facilitating risk communication and shared decision‐making. Importantly, ALNCR is a prognostic marker and does not by itself indicate differential benefit from a specific anticancer therapy; therefore, ALNCR‐guided treatment intensification should be evaluated prospectively before being recommended. In future prospective studies and clinical trials, ALNCR (or ALNCR.TNM) could be incorporated as a baseline stratification factor and/or prognostic covariate to improve balance and statistical efficiency, consistent with regulatory guidance on covariate adjustment and enrichment strategies.

Although the ALNCR.TNM model demonstrated strong predictive power across various cohorts and cancer types, there are several limitations to our study. First, although we included a large cohort of cancer patients, the study population was predominantly from a single institution, which may limit the generalizability of our findings to other healthcare settings. Future studies with diverse, multicentre cohorts are needed to validate the findings in different populations. Second, although our study focused on systemic inflammation as a key predictor, additional molecular and genetic markers should be explored to further refine the ALNCR.TNM model and enhance its prognostic accuracy. Furthermore, although we have established the utility of ALNCR and ALNCR.TNM in predicting survival, additional studies are necessary to assess their potential role in guiding treatment decisions. For example, integrating ALNCR with treatment response markers could help personalize therapeutic approaches, particularly in immunotherapy and targeted therapy.

## 5 Conclusions

In conclusion, the ALNCR.TNM model represents a significant advancement in cancer prognosis by combining systemic inflammation with traditional tumour staging. This model enhances prognostic accuracy, provides a practical tool for clinical risk assessment and has broad applicability across various cancer types. The development of nomograms and web‐based calculators further improves the clinical utility of the ALNCR.TNM model, offering a personalized approach to cancer management. With further validation, this model has the potential to become an integral component of clinical decision‐making in oncology, guiding treatment strategies and improving patient outcomes.

## Funding

This work was supported by the National Key Research and Development Program (2022YFC2009600 and 2022YFC2009601); the Laboratory for Clinical Medicine, Capital Medical University (2023‐SYJCLC01); and the National Natural Science Foundation of China (82404061).

## Ethics Statement

This study followed the Helsinki declaration. All participants signed an informed consent form and this study was approved by the Institutional Review Board of each hospital (Registration number: ChiCTR1800020329).

## Conflicts of Interest

The authors declare no conflicts of interest.

## Supporting information


**Figure S1:** Inflammation‐based indices evaluated in this study.
**Figure S2:** Flow chart.
**Figure S3:** Time‐dependent AUC for predicting overall survival across cohorts.
**Figure S4:** Cutoff value of ALNCR index.
**Figure S5:** Kaplan–Meier overall survival curves stratified by ALNCR across TNM stages and major cancer types.
**Figure S6:** The relationship between the ALNCR index and overall survival among cancer patients.
**Figure S7:** Subgroup Analysis of ALNCR Prognostic Value.
**Figure S8:** The distribution of ALNCR index in different TNM stage.
**Figure S9:** The distribution of ALNCR index in different tumour type.
**Figure S10:** Time‐dependent AUC comparing ALNCR.TNM, ALNCR, and TNM stage in different cohorts.
**Figure S11:** Predictive performance of the ALNCR‐TNM model compared to TNM stage and ALNCR.
**Figure S12:** Clinical Utility and Validation of the ALNCR‐TNM Model.
**Figure S13:** Clinical Utility and Validation of the ALNCR‐TNM Model.
**Table S1:** Inflammatory biomarkers evaluated in this study.
**Table S2:** Discriminative performance of inflammatory indices for overall survival.
**Table S3:** Multivariable Cox regression of the individual components of ALNCR across cohorts.
**Table S4:** The sensitivity analysis of the relationship between ALNCR index and survival.
**Table S5:** Model discrimination between ALNCR, TNM stage, and ALNCR.TNM.

## Data Availability

The datasets used and/or analysed during the current study are available from the corresponding author on reasonable request.
