# Supplementary material for: ALNCR.TNM as a Novel Prognostic Tool for Cancer Survival
Source: J Cachexia Sarcopenia Muscle. 2026 Aug 18;17(4):e70339. doi: 10.1002/jcsm.70339 (PMC13487379; doi:10.1002/jcsm.70339)
Supplement: Supplementary file 1 [file JCSM-17-e70339-s001.docx]

**Figure S1. Inflammation-based indices evaluated in this study.**

**Figure S2.** **Flow chart.**

**Figure S3. Time-dependent AUC for predicting overall survival across cohorts.**

**Figure S4. Cutoff value of ALNCR index.**

Notes: ALNCR, Albumin-Lymphocyte-Neutrophil-C-reactive protein ratio.

**Figure S5. Kaplan–Meier overall survival curves stratified by ALNCR across TNM stages and major cancer types.**

A. Stage I Patients. B. Stage II Patients. C. Stage III Patients. D. Stage IV Patients. E. Lung cancer. F. Esophageal cancer. G. Gastric cancer. H. Liver cancer. I. Pancreatic cancer. J. Colorectal cancer. K. Breast cancer. L. Urinary system tumors.

**Figure S6. The relationship between the ALNCR index and overall survival among cancer patients.**

A. Internal Training Cohort. B. Internal Validation Cohort. C. External Validation Cohort. D-F. Model a-c. Notes: Model a: No adjusted. Model b: Adjusted for age, sex, BMI, TNM stage. Model c: Adjusted for age, sex, BMI, TNM stage, tumor types, cachexia, surgery, coronary heart disease, radiotherapy, chemotherapy, hypertension, diabetes, smoking, drinking, family history.

**Figure S7. Subgroup Analysis of ALNCR Prognostic Value.**

Notes: The model adjusted for age, sex, BMI, TNM stage, tumor types, surgery, radiotherapy, chemotherapy, hypertension, diabetes, smoking, drinking, family history.

**Figure S8. The distribution of ALNCR index in different TNM stage.**

**Figure S9. The distribution of ALNCR index in different tumor type.**

**Figure S10. Time-dependent AUC comparing ALNCR.TNM, ALNCR, and TNM stage in different cohorts.**

**Figure S11.** **Predictive performance of the ALNCR-TNM model compared to TNM stage and ALNCR**

CI, confidence interval; NRI, net reclassification index; IDI, integrated discrimination improvement

**Figure S12. Clinical Utility and Validation of the ALNCR-TNM Model**

**Figure S13. Clinical Utility and Validation of the ALNCR-TNM Model**

**Table S1. Inflammatory biomarkers evaluated in this study.**

| Biomarker name | Biomarker formulas |
| --- | --- |
| Neutrophil-to-Lymphocyte ratio (NLR) | Neutrophil (/µL) / lymphocyte (/µL) |
| Platelet-to-Lymphocyte ratio (PLR) | Platelet (/µL) / lymphocyte (/µL) |
| C-reactive protein-to-albumin ratio (CAR) | C-reactive protein (mg/dL) / albumin (g/dL) |
| Systemic-Immune-Inflammation Index (SII) | Platelet (/µL) × Neutrophil (/µL) / lymphocyte (/µL) |
| Prognostic Nutritional Index (PNI) | 10 × Albumin (g/dL) + 0.005 × lymphocyte (/µL) |
| **Albumin-Lymphocyte-Neutrophil-C-reactive protein ratio (ALNCR)** | **(Albumin (g/dL) × lymphocyte (/µL)) / (Neutrophil (/µL) × C-reactive protein (mg/dL))** |

**Table S2. Discriminative performance of inflammatory indices for overall survival.**

| **Internal training cohort** | | | | **Internal validation cohort** | | | | | **External validation cohort** | | | | |
| --- | --- | --- | --- | --- | --- | --- | --- | --- | --- | --- | --- | --- | --- |
|  | **c-index** | **lower.95** | **upper.95** |  | **c-index** | | **lower.95** | **upper.95** |  | **c-index** | | **lower.95** | **upper.95** |
| **ALNCR** | 0.645 | 0.631 | 0.658 | **ALNCR** | | 0.666 | 0.645 | 0.686 | **ALNCR** | | 0.657 | 0.624 | 0.690 |
| **CAR** | 0.636 | 0.622 | 0.649 | **CAR** | | 0.650 | 0.629 | 0.671 | **CAR** | | 0.644 | 0.611 | 0.677 |
| **PNI** | 0.613 | 0.601 | 0.629 | **PNI** | | 0.614 | 0.592 | 0.636 | **PNI** | | 0.612 | 0.579 | 0.648 |
| **NLR** | 0.612 | 0.598 | 0.625 | **NLR** | | 0.640 | 0.619 | 0.661 | **NLR** | | 0.625 | 0.591 | 0.659 |
| **SII** | 0.596 | 0.582 | 0.611 | **SII** | | 0.635 | 0.614 | 0.657 | **SII** | | 0.596 | 0.563 | 0.630 |
| **PLR** | 0.563 | 0.549 | 0.578 | **PLR** | | 0.568 | 0.546 | 0.590 | **PLR** | | 0.556 | 0.521 | 0.591 |
| **CRP** | 0.631 | 0.618 | 0.645 | **CRP** | | 0.646 | 0.625 | 0.668 | **CRP** | | 0.636 | 0.602 | 0.669 |
| **Albumin** | 0.613 | 0.599 | 0.627 | **Albumin** | | 0.620 | 0.598 | 0.642 | **Albumin** | | 0.613 | 0.578 | 0.648 |
| **Neutrophils** | 0.586 | 0.572 | 0.600 | **Neutrophils** | | 0.637 | 0.616 | 0.658 | **Neutrophils** | | 0.596 | 0.562 | 0.630 |
| **Lymphocyte** | 0.568 | 0.554 | 0.583 | **Lymphocyte** | | 0.544 | 0.522 | 0.566 | **Lymphocyte** | | 0.582 | 0.547 | 0.618 |
| **PLT** | 0.520 | 0.505 | 0.534 | **PLT** | | 0.551 | 0.528 | 0.573 | **PLT** | | 0.494 | 0.459 | 0.529 |

**Table S3. Multivariable Cox regression of the individual components of ALNCR across cohorts.**

|  | **Internal training cohort**  **n=** **4464** | | **Internal validation cohort**  **n=** **1910** | | **External validation cohort**  **n=** **759** | |
| --- | --- | --- | --- | --- | --- | --- |
| **Variable** | **HR (95% CI)** | **P-value** | **HR (95% CI)** | **P-value** | **HR (95% CI)** | **P-value** |
| **Albumin (log, per SD)** | 0.91 (0.87-0.94) | <0.001 | 0.93 (0.85-1.02) | 0.135 | 0.88 (0.77-1.01) | 0.08 |
| **Lymphocyte (log, per SD)** | 0.92 (0.88-0.96) | <0.001 | 0.96 (0.89-1.03) | 0.237 | 0.86 (0.76-0.98) | 0.021 |
| **Neutrophils (log, per SD)** | 1.08 (1.02-1.13) | 0.004 | 1.20 (1.11-1.30) | <0.001 | 1.04 (0.91-1.20) | 0.574 |
| **CRP (log, per SD)** | 1.19 (1.13-1.26) | <0.001 | 1.31 (1.20-1.42) | <0.001 | 1.30 (1.13-1.50) | <0.001 |

**Table S4. The sensitivity analysis of the relationship between ALNCR index and survival.**

| **No exclusion analysis** | | | | | | |
| --- | --- | --- | --- | --- | --- | --- |
| ALNCR | Model a | p value | Model b | p value | Model c | p value |
| Continuous (per SD) | 0.195(0.134, 0.284) | <0.001 | 0.440 (0.320,0.603) | <0.001 | 0.512 (0.380,0.690) | <0.001 |
| Cutoff value |  | <0.001 |  | <0.001 |  | <0.001 |
| C1 (<3.21) | ref |  | ref |  | ref |  |
| C2 (≥3.21） | 0.422 (0.385,0.464) |  | 0.558 (0.507,0.614) |  | 0.594 (0.539,0.655) |  |
| Quartiles |  |  |  |  |  |  |
| Q1 (<0.56) | ref |  | ref |  | ref |  |
| Q2 (0.56,3.47) | 0.713 (0.637,0.800) | <0.001 | 0.739 (0.659,0.829) | <0.001 | 0.757 (0.674,0.851) | <0.001 |
| Q3 (3.47,10.20) | 0.420 (0.370,0.477) | <0.001 | 0.523 (0.460,0.595) | <0.001 | 0.548 (0.480,0.626) | <0.001 |
| Q4 (≥10.20) | 0.311 (0.272,0.356) | <0.001 | 0.453 (0.395,0.519) | <0.001 | 0.497 (0.432,0.572) | <0.001 |
| p for trend |  | <0.001 |  | <0.001 |  | <0.001 |
|  | | | | | | |
| **Excluding** **patients with short-term deaths** | | | | | | |
| ALNCR | Model a | p value | Model b | p value | Model c | p value |
| Continuous (per SD) | 0.989 (0.985,0.992) | <0.001 | 0.994 (0.992,0.997) | <0.001 | 0.996 (0.993,0.998) | 0.031 |
| Cutoff value |  | <0.001 |  | <0.001 |  | <0.001 |
| C1 (<3.21) | ref |  | ref |  | ref |  |
| C2 (≥3.21） | 0.474 (0.428,0.523) |  | 0.618 (0.558,0.684) |  | 0.657 (0.593,0.729) |  |
| Quartiles |  |  |  |  |  |  |
| Q1 (<0.56) | ref |  | ref |  | ref |  |
| Q2 (0.56,3.47) | 0.760 (0.670,0.862) | <0.001 | 0.796 (0.702,0.904) | <0.001 | 0.806 (0.709,0.916) | <0.001 |
| Q3 (3.47,10.20) | 0.528 (0.461,0.605) | <0.001 | 0.647 (0.564,0.742) | <0.001 | 0.670 (0.583,0.771) | <0.001 |
| Q4 (≥10.20) | 0.365 (0.316,0.422) | <0.001 | 0.525 (0.453,0.608) | <0.001 | 0.577 (0.496,0.671) | <0.001 |
| p for trend |  | <0.001 |  | <0.001 |  | <0.001 |

| **Excluding patients with coronary heart disease** | | | | | | |
| --- | --- | --- | --- | --- | --- | --- |
| ALNCR | Model a | p value | Model b | p value | Model c | p value |
| Continuous (per SD) | 0.986 (0.982,0.989) | <0.001 | 0.993 (0.990,0.996) | <0.001 | 0.994 (0.992,0.997) | <0.001 |
| Cutoff value |  | <0.001 |  | <0.001 |  | <0.001 |
| C1 (<3.21) | ref |  | ref |  | ref |  |
| C2 (≥3.21） | 0.426 (0.387,0.469) | <0.001 | 0.569 (0.516,0.627) | <0.001 | 0.603 (0.546,0.667) |  |
| Quartiles |  |  |  |  |  |  |
| Q1 (<0.56) | ref |  | ref |  | ref |  |
| Q2 (0.56,3.47) | 0.710 (0.632,0.798) | <0.001 | 0.752 (0.669,0.846) | <0.001 | 0.764 (0.678,0.860) | <0.001 |
| Q3 (3.47,10.20) | 0.421 (0.370,0.480) | <0.001 | 0.531 (0.465,0.606) | <0.001 | 0.554 (0.484,0.634) | <0.001 |
| Q4 (≥10.20) | 0.312 (0.272,0.358) | <0.001 | 0.466 (0.405,0.537) | <0.001 | 0.507 (0.439,0.585) | <0.001 |
| p for trend |  | <0.001 |  | <0.001 |  | <0.001 |

Notes:

Model a: No adjusted.

Model b: Adjusted for age, sex, BMI, TNM stage.

Model c: Adjusted for age, sex, BMI, TNM stage, tumor types, surgery, radiotherapy, chemotherapy, hypertension, diabetes, smoking, drinking, cachexia, family history.

**Table S5. Model discrimination between ALNCR, TNM stage, and ALNCR.TNM.**

| **Internal training cohort** | | | | **Internal validation cohort** | | | | **External validation cohort** | | | |
| --- | --- | --- | --- | --- | --- | --- | --- | --- | --- | --- | --- |
|  | c-index | Lower 95 | Upper 95 |  | c-index | lower 95 | upper 95 |  | c-index | lower 95 | upper 95 |
| ALNCR | 0.613 | 0.601 | 0.624 | ALNCR | 0.628 | 0.610 | 0.645 | ALNCR | 0.623 | 0.596 | 0.649 |
| TNM stage | 0.612 | 0.603 | 0.621 | TNM stage | 0.612 | 0.598 | 0.626 | TNM stage | 0.574 | 0.558 | 0.591 |
| ALNCR.TNM | 0.675 | 0.664 | 0.687 | ALNCR.TNM | 0.687 | 0.669 | 0.704 | ALNCR.TNM | 0.658 | 0.632 | 0.684 |
